# Robust multiple-scroll dynamics in memristive-based generator system

**DOI:** 10.1038/s41598-023-34423-1

**Published:** 2023-05-22

**Authors:** A. O. Adelakun, Y. A. Odusote

**Affiliations:** Department of Physics, Federal University of Technology, Akure, Ondo state Nigeria

**Keywords:** Mathematics and computing, Physics

## Abstract

In-depth analysis of a novel multiple scroll memristive-based hyperchaotic system with no equilibrium is provided in this work. We identify a family of more complicated $n\mathrm{th}$-order multiple scroll hidden attractors for a unique, enhanced 4-dimensional Sprott-A system. The system is particularly sensitive to initial conditions with coexistence and multistability of attractors when changing the associated parameters and the finite transient simulation time. The complexity (CO), spectral entropy (SE) algorithms, and 0-1 complexity characteristics was thoroughly discussed. On the other hand, the outcomes of the electronic simulation are validated by theoretical calculations and numerical simulations.

## Introduction

For decades, the dynamical transition of system from periodic state to chaos and hyperchaos has attracted a great interest and majorly considered as an active area of research with great practical significance. The sensitive dependence is among the basic ideas of chaos and most visible signature of its behavior. Several studies have shown that rich dynamics can be deduce from improved systems by introducing new terms, changes to initial conditions, increasing the dimension, and nonlinearity type^1^. In this regard, an hyperchaotic system is defined as a chaotic system with at least two positive Lyapunov exponents, high sensitivity to initial conditions, more randomness, higher unpredictability and at least four dimensional phase space. Obviously, hyperchaotic systems have wide applications and therefore more preferred than the chaotic systems in recent central topic researches including synchronization^2^, neural networks^3^, finance^4^, nonlinear circuits^2–5^, chaos-based secure communication^28^ among others. Such systems exhibits multi-scroll and multi-wings attractors and therefore played a vital role in engineering and technology applications^6–8^.

Thus, comes the idea to classify the hyperchaotic systems based on their performance and optimization purpose. Such special systems have been classified based on number and types of equilibrium which includes: (1) hidden attractors with one equilibrium, (2) hidden attractors with line of infinite equilibria, and (3) hidden attractors without equilibria or conjugate equilibria^9^. These systems contradict the Shilnikov criteria, which requires at least one unstable equilibrium for the emergence of chaos^10^. Accordingly, systems with equilibrium points are purely based on stability criterion, connection with criteria as well as the theorems that determine the existence of chaos in Shilnikov chaos, Melnikov function to mention a few^11,12^. Loss of equilibrium point, however, is an indication that no conventional Shilnikov criteria can be applied to investigate the flow of chaos^9^. Likewise, it has been established that the basin of attraction does not intersect with small neighborhoods of any equilibrium points^13^. As seldomly reported, systems without equilibrium usually give birth to hidden attractors, which implies that self-excited attractors can be derived from unstable equilibrium if no intersection occurs between the basin of attraction and in any open neighborhoods of a stationary state^14–16^. Actually, systems with a line of equilibria^17^ and infinite equilibria^18^ have also been modified into systems without equilibrium points by adding a simple parameter.

Since the discovery of a simple flow system with no equilibrium points^19^, more attention on the dynamical behavior and expected application of multi-scroll hidden attractors have been received. For example, a special case of Nose-Hoover oscillator “Sprott-A” that has features of been boosted by his state variables has been investigated^20^. Therefore, changing the DC offset of the variable to any level is said to be conservative and has no equilibrium points^9,11,21^. Interestingly, the application of such spellbinding hyperchaotic system is therefore very vital in engineering when transforming bipolar signal to unipolar signal and vice versa^22,23^. Other reported modifications to Sprott A systems without equilibrium in recent years include, (1) replacing the $y^2$ term with a new $\vert y \vert$^24^; and (2) *x* with cubic nonlinearity term^9^. In addition, Sprott-D system with a perturbation term to non-hyperbolic equilibrium also gives a system with hyperchaotic features even in the absence of no equilibrium points^25^. So far, it has been confirmed that some hyperchaotic systems that exhibit multi-scroll and multi-wing behaviors have also been proven to carry more complexity than those with few attractors^26^. These new findings will play a major role and robust application in image encryption^26^, memristors^27^, and secure communication devices^28^. Very recently, Jafari et al.^26^ proposed a 2 by 3 grid multi-scroll and multi-wing attractors with hidden attractors from 3D-system without equilibrium by simple state variable modification to the original Sprott-A system. Hu et al.^29^ also discovered a multi-scroll chaotic sea in a simple sine function nonlinear 3D Sprott-A system, while a numerical and experimental validation of an improved Jerk hyperchaotic multi-scroll system has been reported with infinite number of equilibrium^28^. Systems without equilibrium, hidden attractors that exhibits multi-scroll and multi-wings has also been discovered in the Lorenz-like system with a designed saw tooth wave function 4D system using a state feedback controller^30^.

Meanwhile, a safer communication key can be produced by the memristor’s high complexity in hiding the chaos, which has major theoretical implications for the advancement of chaotic security technology. Due to its low-power processors, high-speed, associative memory, adaptive filter, pattern recognition systems, programmable analog integrated circuits, and neural networks, the development of memristor as a replacement for Chua’s diode also supports prospective memristor-based applications^24,31,32^. Therefore multiple dynamics can coexist simultaneously in the operation of memristor and the dynamics of a multistable system. These systems were extremely sensitive to initial conditions thereby generating to what is referred to as multi-stability of hidden attractors due to coexistence of many infinite attractors^33–38^. Inspired with recent findings, we intend to propose a new 4D memristive-based Sprott-A system with multiple hidden attractors from specific control parameters and simple to implement practically. The organization of this paper is as follows: section “Model and methods” gives a mathematical description and the theoretical properties of the proposed model. Section “Results” reports the numerical and electronic simulation results. Section “Concluding remarks” concludes this paper.

## Model and methods

### Memristor-based 4D Hyperchaotic Sprott-A system

Though not all nonlinear systems are complex, nonlinearity creates opportunities for complex behavior that are not available in linear systems^39,40^. In this study, we added a fourth passive component with improved nonlinear characteristics. We shall introduce the memristor $W(\phi )$, a component made up of an electrical charge (q) and a magnetic flux ($\phi$) that has been physically achieved by Stanley William’s team at HP labs^41–47^. Herein, the smooth cubic flux-controlled memristor is hereby characterized by a smooth continuous cubic nonlinearity given by^12^

1 $$\begin{aligned} \left\{ \begin{array}{ll}q(\phi )=\beta \phi +\xi {\phi }^3\\ W(\phi )=\frac{dq(\phi )}{d\phi }=\beta +3\xi {\phi }^2\\ i=W(\phi )v.\end{array}\right. \end{aligned}$$

where $\beta$ and $\xi$ are parameters embedded in the memristive-based function, $\phi$ is the internal state variable of $W(\phi )$. Also note that *v* is the input voltage while $i=f(W,v_2)$ is the output voltage of the memristor $W(\phi )$. The state variables correspond to voltages across each capacitor used in the designed circuit i.e $x=v_1$, $y=v_2$, $z=v_3$, $w=\phi$, $v=v_2$ and *f*(*w*)=$W(\phi )$. It is worth noting that *w* is the internal state of the memristive device. In this section, we examine the main and elementary dynamic properties of the robust memristor-based system (2). The system is constructed by a simple modification to the special case of 3-D Nose-Hoover system, Sprott-A^17^. The system is composed of extended state variable $z \rightarrow z+\alpha sin z$ and the cubic nonlinear function, generally meant to model smooth cubic-flux controlled memristor. The new 4-D system can be expressed as:

2 $$\begin{aligned} \left\{ \begin{array}{ll}{\dot{x}}=ay\\ {\dot{y}}=-x+y[bf(z)+cf(w)]\\ {\dot{z}}=\varepsilon -y^2\\ {\dot{w}}=y.\end{array}\right. \end{aligned}$$

The nonlinear function $f(z)=z+\alpha sinz$ is introduced to explore the multiple dynamical behaviors with slight changes to $\alpha$. The other parameters a, b, c, $\xi$ and $\beta$ are carefully chosen when determining the complexity of system (2). With a suitable parameter and changes to initial conditions, the system can generate family of multi-scroll and multi-wing attractors. For example, the phase portrait of attractors can be plotted by setting: a = 0.05, b = 0.1, c = 0.01, $\alpha =25$, $\varepsilon =1$ $\beta =-6e^{-4}$ and $\xi =5e^{-4}$, *x*(0.1), *y*(0), *z*(0) and *w*(0). System (2) is invariant under the natural coordinates transformation $(x,y,z,w)\mapsto (-x,-y,z,-w)$ and persists for all values of the system parameters.

### Complexity and 0-1 test analyses

In this study, we will quantify the system’s complexity (2) using complexity (CO) and information spectrum analysis (SE). The detailed algorithms has been researched in the literature^48^. The algorithms are a powerful measure of the chaotic properties of the system and may better measure the structural complexity of the high-dimensional hyperchaotic system as a whole as an excellent algorithm in structural complexity. The completeness on the system (2) can be achieved by computing the energy distribution in the Fourier transform domain and combining it with the Shannon entropy (SE), whereas complexity is achieved by decomposing time series into regular and irregular conceptions (C0). The required chosen parameter is based on the time series’ irregular evolution. In order to further characterize the robust dynamical behavior of the new system, we also explore the 0-1 test approach^49^. We used the 0-1 chaos test to assess the dynamic properties of the system (2), which offers information on regular and irregular dynamics embedded in a deterministic system. The input of the test is 1-dimensional time series $\phi$(n) for n=1,2...,N. The time series data is used to derive the 2-dimensional algorithm as follows:

3 $$\begin{aligned} \left\{ \begin{array}{ll} s(n+1)&{}=~s(n)+\phi (n)~cos~(cn)\\ p(n+1)&{}=~q(n)+\phi (n)~sin~(cn).\end{array}\right. \end{aligned}$$

where c $\epsilon$ $(0,2\pi )$ is fixed. The underlying bounded $p-s$ motion reveal the regular and asymptotic brownian motion of the deterministic system. That is, the asymptotic dynamics in the (p,s)-plane of the 2-dimensional Euclidean extensions of a given dynamical system is typically bounded for regular motions and unbounded for non-regular motions.

## Results

### Stability analysis and dynamic properties

Alternatively, the best way of analyzing the proposed system is to determine whether the equilibrium point exists or not. Next is to characterize the local dynamical behavior of the system orbits near these points, which indicates self-excited attractors, otherwise, hidden attractors. Firstly, the equilibria can be obtained by equating the differential part of system (2) to zero.

4 $$\begin{aligned} \left\{ \begin{array}{ll}0=ay\\ 0=-x+y[bf(z)+cf(w)]\\ 0=\alpha -y^2\\ 0=y.\end{array}\right. \end{aligned}$$

However, it is obvious that the system has no solution, which implies that the system is without equilibrium for the chosen parameter value(s). The rate of volume expansion of the system is given as follows;

5 $$\begin{aligned} \nabla V(x,y,z,w)=\frac{\partial {\dot{x}}}{\partial x}+\frac{\partial {\dot{y}}}{\partial y}+\frac{\partial {\dot{z}}}{\partial z}+\frac{\partial {\dot{w}}}{\partial w} =b(z+\alpha sinz)+c \left( \beta +3\xi w^2 \right) \end{aligned}$$

Therefore, for $\nabla =0$, the system is conservative and as long as $\nabla <0$ or $\nabla >0$, then the system is dissipative. For example, if the initial conditions in Table 1 are employed for Eq. (5), the system is clearly dissipative with $\alpha =1$, b = 0.1 and c = 0.01 (say, $\nabla =\beta c$ and $\nabla = b (0.43)+ c (-6e^-5)$), respectively. The adequate information regarding the system clarification on the relationship between different initial conditions will be discussed in the next section with fluctuation across the selected range. This implies that the local volumes in the phase space are contracted exponentially with rate $\beta c$ for hyperchaotic systems at $x_o,y_o,z_o,w_o$ = 0.1, 0, 0, 0 and $b (0.43)+c (-6e^-5)$ for $x_o,y_o,z_o,w_o$ = 0.1, 0.2, 0.3, 0, respectively. Therefore, the systems has an attracting set. We also deduced that the system is dissipative by summing their finite-time local Lyapunov exponents for the selected initial conditions, which are greater than zero (see Table 1). The Kaplan-Yorke fractal dimension, $D_{KY}$, is commonly defined as a fractional dimension in which a cluster of initial conditions will neither expand nor contract as it evolves with time. Then, $D_{KY}$ can be expressed as

6 $$\begin{aligned} D_{KY}=j+\frac{1}{\vert L_{j+1}\vert }\sum _{i=1}^jL_i \end{aligned}$$

where j is the largest integer satisfying $\sum _{i=1}^jL_i\ge 0$ and $\sum _{i=1}^{j+1}L_i< 0$. The Kaplan-Yorke calculation for system (8) is

7 $$\begin{aligned} D_{KY}=3+\frac{L_1+L_2+L_3}{\vert L_4 \vert } \end{aligned}$$

**Table 1:** Proposed dynamic properties for $\varepsilon =1$.

Initial condition	Lyapunov exponents				Dynamic properties		
	$L_1$	$L_2$	$L_3$	$L_4$	$\sum L_{1-4}$	$D_{KY}$	State
0.1,0,0,0	0.0811	0.0085	0.0564	− 0.0973	0.0488	4.5013	Hyperchaotic
0.01,0,0,0	0.1321	0.0456	0.0333	− 0.2133	− 0.0023	3.9891	Hyperchaotic
0.1,0.2,0.3,0	0.1195	0.0217	− 0.0117	− 0.2497	− 0.1202	3.5186	Hyperchaotic
0.1,0.1,0.1,0.1	0.1522	− 0.0373	− 0.0061	0.0165	0.1254	3.8683	Hyperchaotic
− 0.1,0.2,0.3,0.1	0.0859	− 0.0035	− 0.0441	0.0752	0.1135	3.5093	Hyperchaotic
0.2,− 0.2,− 0.2,0	0.0567	− 0.0181	0.0112	− 0.1301	− 0.0803	3.3827	Hyperchaotic
0.001,0.001,0.02,0	1.4886	− 0.0193	− 1.3132	− 0.0728	0.0833	5.1456	Chaotic
0.001,0.001,0.001,0	1.4888	− 0.0214	− 1.2438	− 0.0743	0.1493	6.0094	Chaotic

**Table 2:** Dynamic properties at different values of $\varepsilon$.

$\varepsilon$	Lyapunov exponents				Dynamic properties		
	$L_1$	$L_2$	$L_3$	$L_4$	$\sum L_{1-4}$	$D_{KY}$	State
6	0.1840	− 0.0239	0.1836	− 0.4387	− 0.0950	3.7835	Hyperchaotic
10	0.3906	− 0.0593	0.0598	− 0.3872	0.0039	4.0101	Hyperchaotic
15	0.3064	− 0.0058	0.0034	− 0.3957	− 0.0917	3.7683	Hyperchaotic

In general, sensitivity to initial conditions is one of the determinant factors in specifying the state of chaos in a system. Such response can be traced to chaos-hyperchaos transition or dynamic index measurement for any oscillators. As shown in Table 1, the chaos-hyperchaos properties for different initial conditions are summarized: for finite-time local Lyapunov exponent values ($L_{1-4}$), the summation of Lyapunov exponents ($\sum L$), and Kaplan-Yorke fractals $D_{KY}$. Table 2 shows some theoretical calculations for higher values of $\varepsilon$ that generates number of multi-scroll and multi-wing attractors. Therefore, the proposed system exhibits several hyperchaotic finite-time properties which are based on the variation to initial conditions and some system parameters. Such changes in waveforms indicate chaos and may gives birth to different complex states at time t.

### Bifurcation structures and Lyapunov exponents

**Figure 1:**
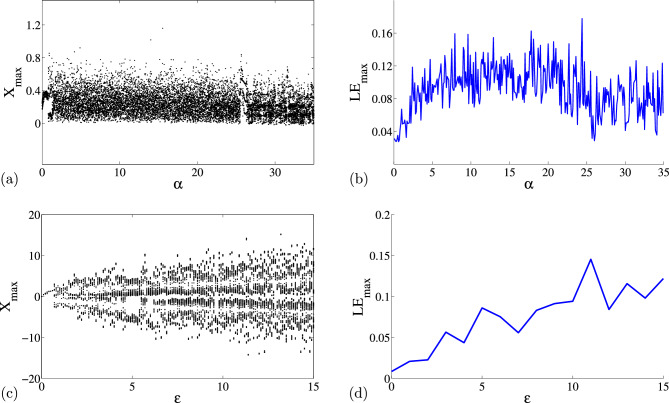
Local (**a**) bifurcation diagram $(\alpha -X_{max})$ and (**b**) corresponding maximum Lyapunov exponents and (**c**) bifurcation diagram $(\varepsilon -X)$ and (**d**) corresponding maximum Lyapunov exponents.

In chaos theory, bifurcation diagrams and Lyapunov exponents plots are used to provide useful illustrations for variations of a particular system dynamics with changes in its parameters. That is, a single system may possess different unstable or stable regions where complex dynamical states can be observed. Small changes to system parameters or modification to system dimensions may lead to a discontinuous flow in the system properties usually referred to as dynamic transition. In this section, the bifurcation diagrams and associated Lyapunov exponents plots from the proposed system (4) is investigated using $4^{th}$-order Runge-Kutta algorithm. The number of multiple scroll can be induced based on changes to different parameters in the system, specifically, $\alpha$, and $\varepsilon$. The bifurcation analysis is of two fold: (1) when varying the parameter $\varepsilon$ and keeping other parameter fixed, and (2) when varying the parameter $\alpha$. In Fig. 1a and b, the $\alpha -X_{max}$ plane plots show the respective robust bifurcation structures and corresponding Lyapunov exponents when the parameter $\alpha$ is varied. The structures simply predict regions of higher dynamical crises, where numbers of scroll can be observed. In addition, weak bifurcation response can be noticed at $\alpha <2.5$, while rich dynamics were observed at $\alpha >2.5$. Similarly, the robust multiple scroll were noticed in $\varepsilon -X$ bifurcation structure and corresponding Lyapunov exponent as displayed in Fig. 1c and d, respectively. For instance, the typical bifurcation and Lyapunov exponent reveal several regions of complexity for $\varepsilon =1$ and $\varepsilon =10$, respectively.

**Figure 2:**
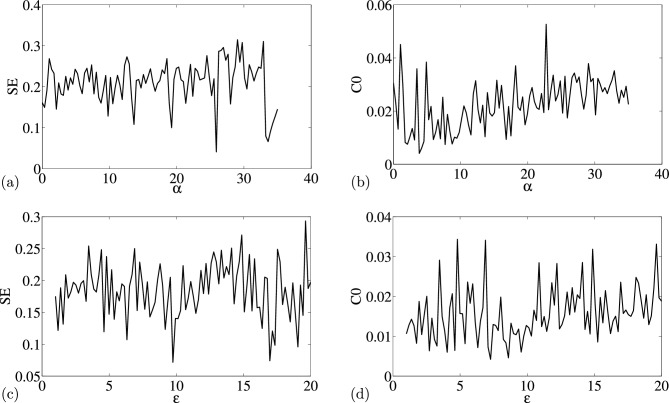
Complexity of the modified Sprott-A system with (i) varying $\alpha$ (**a**) *SE* Complexity and (**b**) *CO* Complexity, and (ii) varying $\varepsilon$, (**c**) *SE* Complexity and (**d**) *C*0 Complexity.

**Figure 3:**
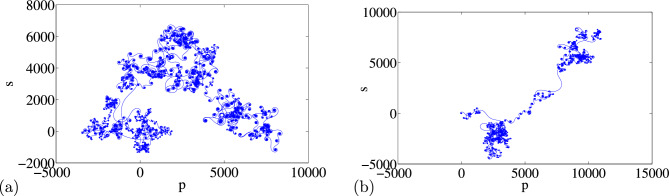
Typical p-s diagram for modified Sprott-A system with 0-1 test for initial conditions: (**a**) $x_0=0.1$,$y_0=0.2$, $z_0=0.3$, $w_0=0$, and (**b**) $x_0=0.1$, $y_0=0$, $z_0=0$ and $w_0=0$,

The information pictured in Figs. 2 and 3 also provide good idea and expected complex dynamical behaviors from the two-parameter plots. The curve displays considerable swings, indicating that changing the parameters $\alpha$ and $\varepsilon$ has a significant impact on structural complexity. Simply by changing the settings across a predetermined range, dynamical behaviors were completely consistent with the attractors. Figure 2a and b shows a wide range of complexity at large value of $\alpha$. The complexity is replicated by the bifurcation diagram and Lyapunov exponent in Fig. 1a and b, respectively. When compared complexity in Fig. 2c and d to the bifurcation diagram and corresponding Lyapunov exponent in Fig. 1c and d, a similar pattern can also be observed. Therefore, in contrast to SE complexity, C0 complexity is accurate and reliable, particularly when a bifurcation point changes. We also noticed anomalies when different initial circumstances are applied to system (2) (see Fig. 3). In Fig. 3a and b, we show how the 0-1 test is able to detect irregular orbits across $p-n$ plane, even in the chaotic case (a) $x_0=0.1$, $y_0=0.2$, $z_0=0.3$, $w_0=0$ and hyperchaotic case (b) $x_0=0.1$, $y_0=0$, $z_0=0$ and $w_0=0$.

### Phase portraits of Attractors and Poincáre cross-section

**Figure 4:**
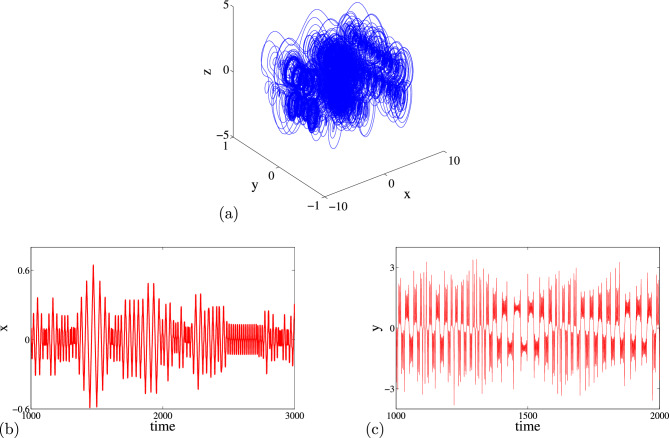
Typical plots for (**a**) modified new Sprott-A attractor on x-y-z plane, (**b**) x vs t time series and (**c**) y vs t time series.

**Figure 5:**
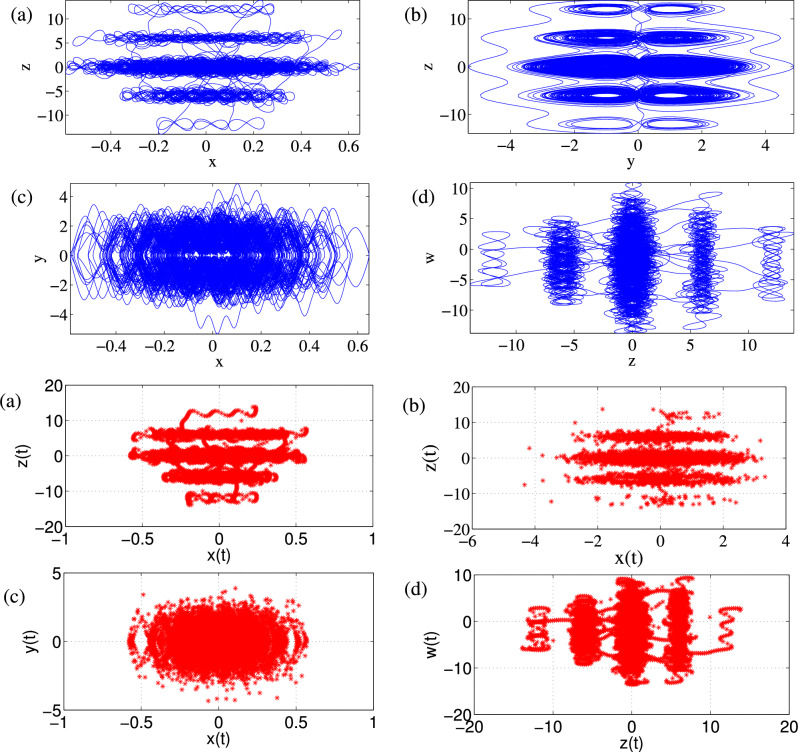
The observatory Multi-scroll attractors and corresponding Poincáre maps for (**a**) z–x, (**b**) z–y, (**c**) y–x and (**d**) w–z with initial condition: 0.1, 0, 0, 0, $\alpha =25$ and t=6000s.

**Figure 6:**
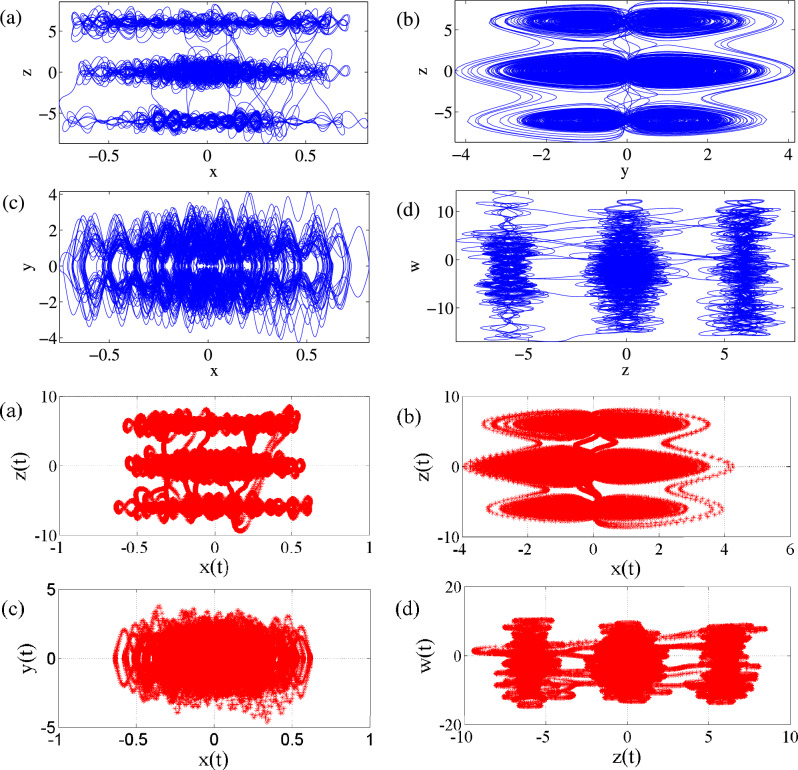
The observatory Multi-scroll attractors and corresponding Poincáre maps for (**a**) z–x, (**b**) z–y, (**c**) y–x and (**d**) w–z with initial condition: 0.1, 0.2, 0.3, 0, $\alpha =25$ and t = 6000 s.

**Figure 7:**
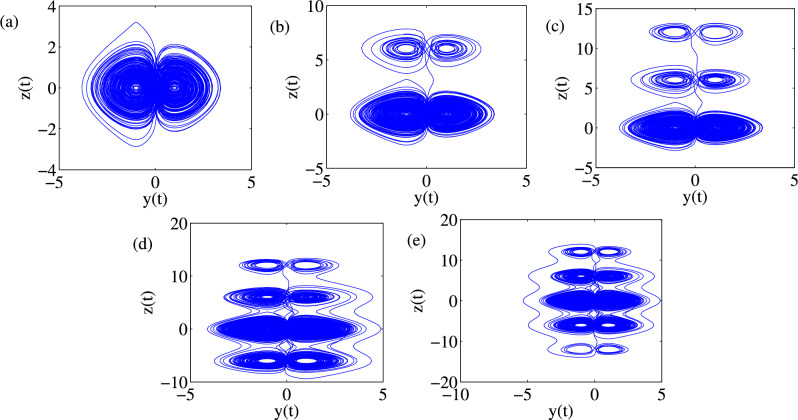
Typical multi-scroll transitions: (**a**) 1-double-scroll and 2-wings (1 $\times$ 2 grid) at t = 1000 s, (**b**) 2-double-scroll and 4-wings (2 $\times$ 2 grids) at t = 1330 s, (**c**) 3-double-scroll and 6-wings (2 $\times$ 3 grids) at t = 1400 s, (**d**) 4-double-scroll and 8-wings (2 $\times$ 4 grids) at t = 5000 s and (**e**) 5-double-scroll and 10-wings (2 $\times$ 5)at $t\ge 5000s$ with initial condition: (0.1,0,0,0) and at $\alpha =25$.

**Figure 8:**
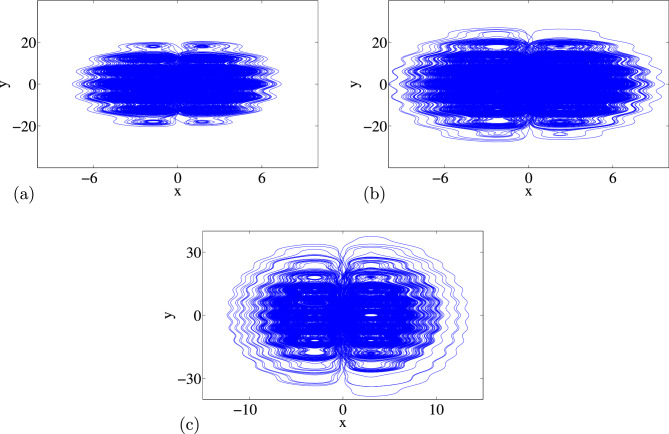
Typical multi-scroll and (**a**) seven-wings at $\varepsilon =6$, (**b**) nine-wings at $\varepsilon =10$, (**c**) eleven-wings at $\varepsilon =15$ with initial condition: 0.1,0,0,0 at time t = 20,000.

**Figure 9:**
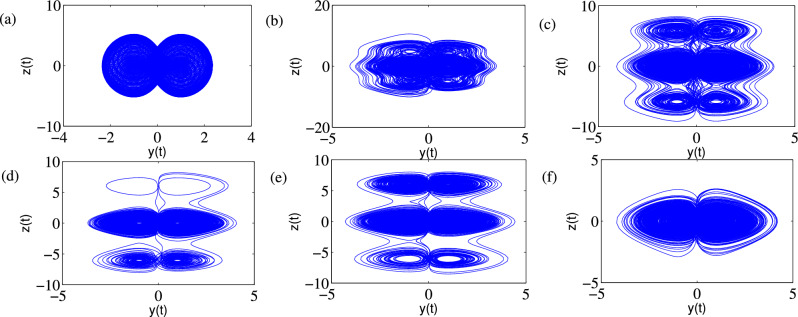
Typical multi-scroll transitions: (**a**) two-scroll torus (1 $\times$ 2 grid) at $\alpha =0$, (**b**) (2 $\times$ 3 grid) chaotic at $\alpha =5$, (**c**) 3-double-scroll and 6-wings (2 $\times$ 3 grids) at $\alpha =15$, (**d**) 3-double-scroll and 6-wings (2 $\times$ 3 grids) at $\alpha =26$ and (**e**) 3-double-scroll and 6-wings (2 $\times$ 3 grids) at $\alpha =30$ and (**f**) two-scroll chaotic (1 $\times$ 2 grid) at $\alpha =34$ with initial condition: (0.1,0.2,0.3,0).

Feasibility of the arbitrary number of multi-scroll and multi-wing hidden attractors from the new modified Sprott-A system were obtained by varying the parameter $\alpha$ and when simulation time t increases at $\varepsilon =1$. However, n-scroll and butterfly wings can also be produced by setting $\varepsilon >1$. From the phase portrait in Fig. 4a, the typical 3D view of the new proposed hyperchaotic 4D Sprott A-type is plotted, while Fig. 4b and c are the time series for the $x-t$ and $y-t$ planes, respectively. However, number of multi-scroll and multi-wing hidden attractors and corresponding Poincáre maps could be generated from: (1) hyperchaotic with initial condition: $(x_0=0.1,y_0=0,z_0=0,w_0=0)$ (see Fig.5a–d), and (2) chaotic with initial condition $(x_0=0.1,y_0=0.2,z_0=0.3,w_0=0)$ as shown in Fig. 6a–d. Figure 7 shows the transition to bigger number of multi-scroll and multi-wing hidden attractors for the hyperchaotic system when simulation time increases. Meanwhile, further studies on n-scroll attractors were plotted when $\varepsilon >1$ as shown in Fig. 8. For instance, when $\varepsilon =6$, the $2\times 7$ grid is produced. Similarly, for $\varepsilon =10$ and $\varepsilon =15$ depicts to $2\times 9$ grid and $2\times 11$ grid were generated, respectively. In addition, birth and death of multiple hidden attractors were also developed when varying the parameter, $\alpha$, (see Fig. 9) as reported in hyperchaotic bifurcation diagram and Lyapunov exponent in Fig. 1a and b.

### Experimental realization

**Figure 10:**
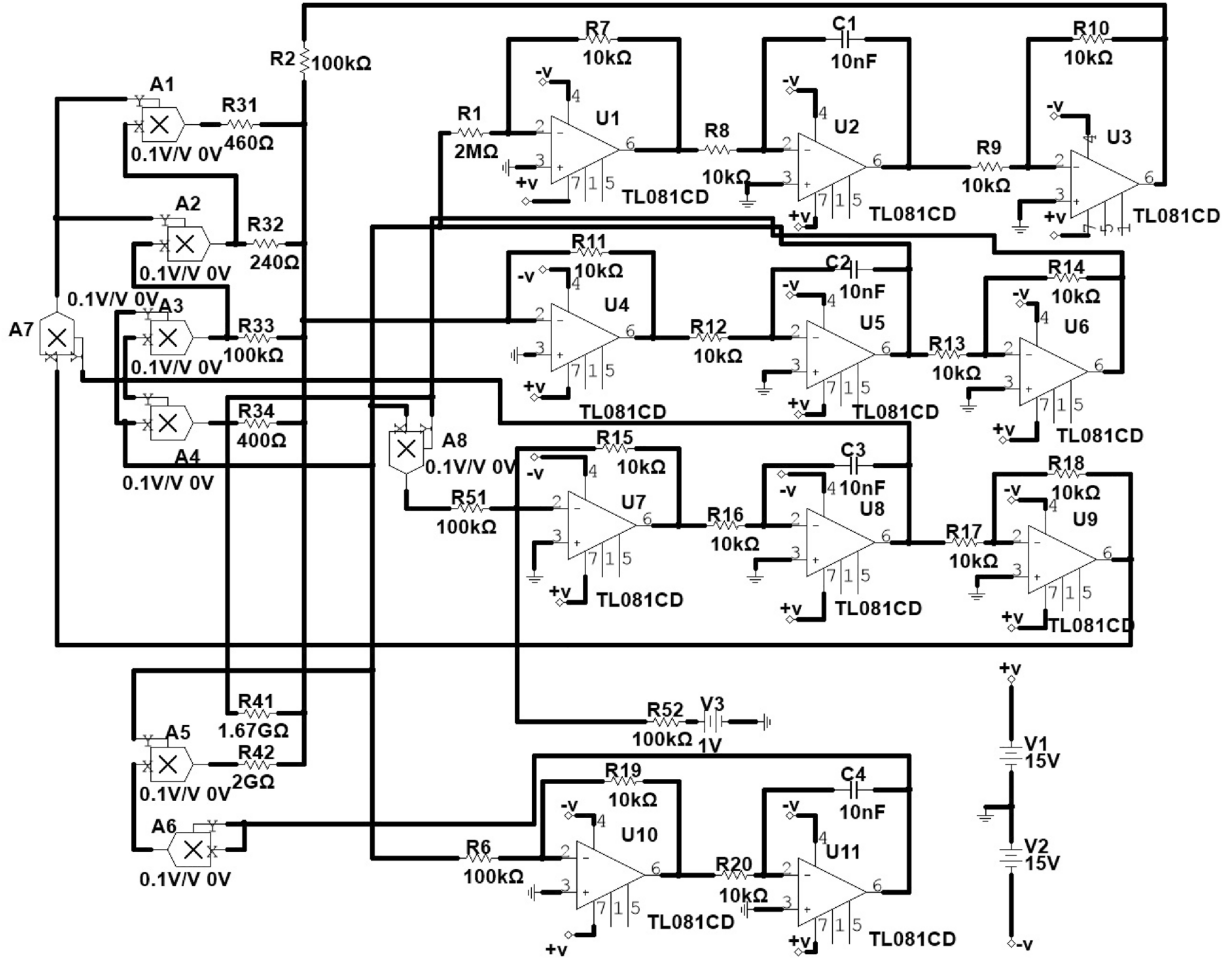
Experimental circuit diagram.

To further verify the correctness of the proposed multi-scroll and multi-wing hyperchaotic system with hidden attractors, the corresponding analog circuit experiment is carried out in this section. The exact circuitry realization of the proposed system and the simulation results must validate the numerical and theoretical findings after properly chosen circuitry parameters. We design and implement the circuit topology on the Pspice breadboard, and the results are presented accordingly. The set-up is displayed in Fig. 10. The analog implementation of the differential form in Eq. (2) is carried out with trigonometry sine function, operational amplifier (TL081CD), multiplier (AD633JN), power supply at $\pm 15V$. The circuit equation can be expressed as; where the transformed function $f(v_z)=v_z+ \alpha sin(v_z)$ and the memristive function $f(v_\phi )=W(\phi )=-\beta +3\xi v_\phi ^2$, $v_x=x$, $v_y=y$, $v_z=z$ and $v_{\phi }=w$. The state variables $v_x$, $v_y$, $v_z$, and $v_\phi$ are associated accordingly with the voltages across the proposed system.

**Figure 11:**
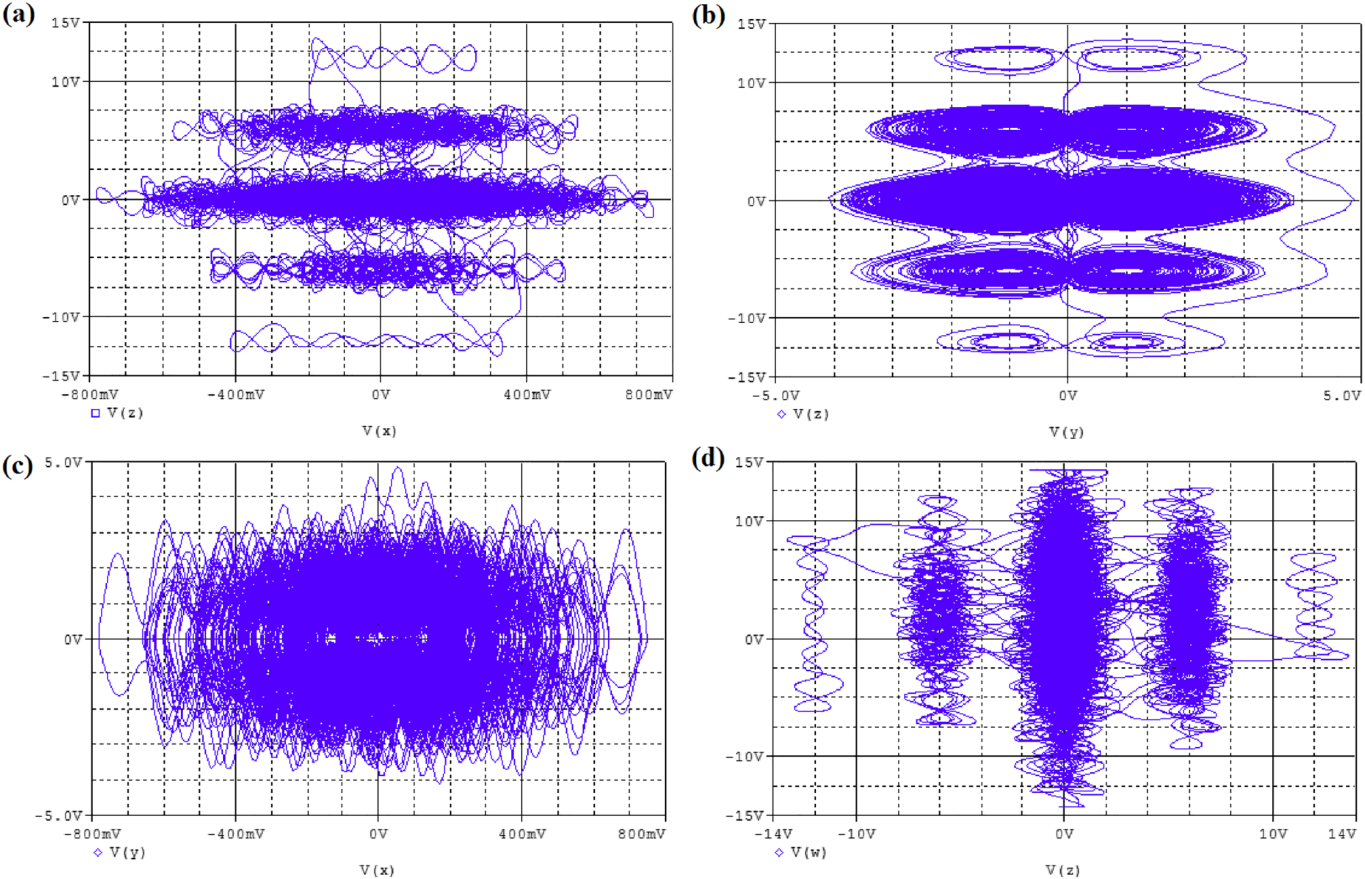
The simulation phase portraits attractor for hyperchaotic oscillator with initial condition, $x_o=0.1,y_o=0,z_o=0,w_o=0$, in blue color: (**a**) $V_z$ vs $V_x$, (**b**) $V_z$ vs $V_y$, (**c**) $V_y$ vs $V_x$ and (**d**) $V_w$ vs $V_z$, respectively at $\alpha =25$.

**Figure 12:**
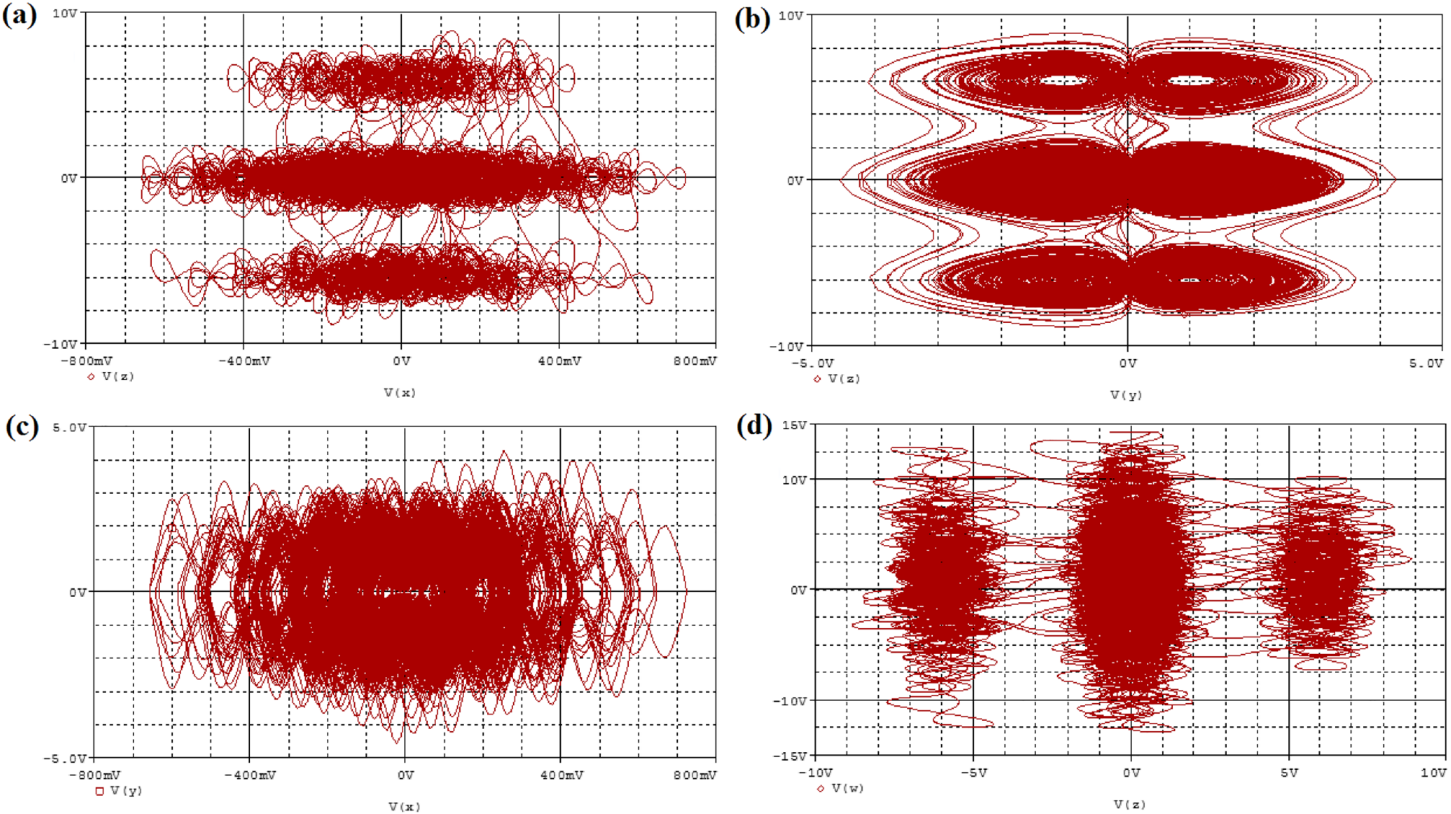
The simulation phase portraits attractor for hyperchaotic oscillator with initial condition ($x_o=0.1,y_o=0.2,z_o=0.3,w_o=0$) in brown color: (**a**) $V_z$ vs $V_x$, (**b**) $V_z$ vs $V_y$, (**c**) $V_y$ vs $V_x$ and (**d**) $V_w$ vs $V_z$, respectively at $\alpha =25$.

**Figure 13:**
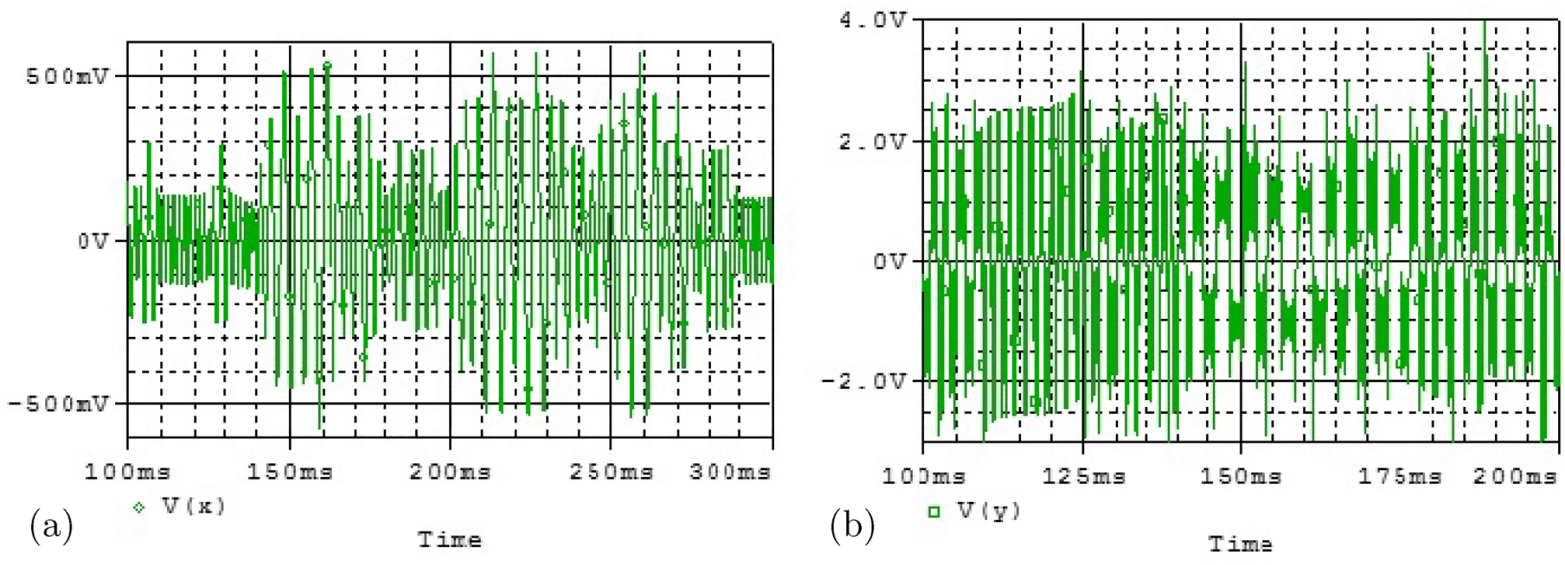
Typical time series plots, $V_x$ and $V_y$ against time at $\alpha =25$.

The electronic simulation results in Fig. 11 is consistent with the numerical simulation results in Fig. 5 for initial condition $x_0=0.1,y_0=0.2,z_0=0.3,w_0=0$. The electronic version in Fig. 12 also validate the numerical simulation in Fig. 6 of initial condition: $x_0=0.1,y_0=0,z_0=0,w_0=0$. The electronic time series version of $V_x$ and $V_y$ against time were plotted in Fig. 13a and b, respectively. Finally, the memristor-based topology with multiple attractors is likely convenient to boost the chaos generation and found important application in biometric authentification, broadband signal generators, pseudo-random number generators, synchronization as well as secure communication.

## Concluding remarks

In summary, we have examined and analyzed the robust hyperchaotic behaviors emanated from a new modified memristive-based 4D Sprott-A system. The new proposed model reveals no equilibrium point, sensitive to initial conditions, and transition from chaos to hyperchaos state with coexistence and multistability of attractors. Various multi-scroll and multi-wings attractors from the proposed system have been deduced from a control parameter. We also discovered time variance hyperchaotic hidden multi-scroll and multi-wing phase portraits when varying a number of scroll determinant parameter $\alpha$. The n-scroll multiple scroll generator is also reported when the parameter $\varepsilon$ is changed. The comparative results convincingly show that the complicated irregulaties within the selected parameters are shown by SE complexity, C0 complexity, and $p-s$ motion. Finally, there is excellent agreement between the calculated results and the observed results from the actualized electrical circuit.

## Data Availability

No data were created or analyzed in this study.
